# The genome sequence of *Tethysbaena scabra* (Pretus, 1991), the first known in the peracarid crustacean order *Thermosbaenacea*.

**DOI:** 10.12688/f1000research.161461.2

**Published:** 2025-07-04

**Authors:** Joan Pons, Karen D. Schöninger-Almaraz, Laura Triginer-Llabrés, Carlos Juan, Damià Jaume, José A. Jurado-Rivera

**Affiliations:** 1 Animal and Microbial Biodiversity, Institut Mediterrani d'Estudis Avancats, Esporles, Illes Balears, 07190, Spain; 2 Centre Balear de Biodiversitat, Departament de Biologia, Universitat de les Illes Balears, Palma, Balearic Islands, 07122, Spain; 3 Biologia, Universitat de les Illes Balears, Palma, Balearic Islands, 07122, Spain

**Keywords:** Thermosbaenacea, anchialine environment, stygobiont species, Tethysbaena scabra

## Abstract

We present a genome assembly of *Tethysbaena scabra* (Arthropoda; Crustacea; Malacostraca; Eumalacostraca; Peracarida; Thermosbaenacea; Monodellidae), a species endemic to Mallorca, Spain. The genome size is 1.18 gigabases that is scaffolded into 17 chromosomes plus a mitochondrial genome of 16,5 kilobases in length.

## Introduction

**Figure 1.**
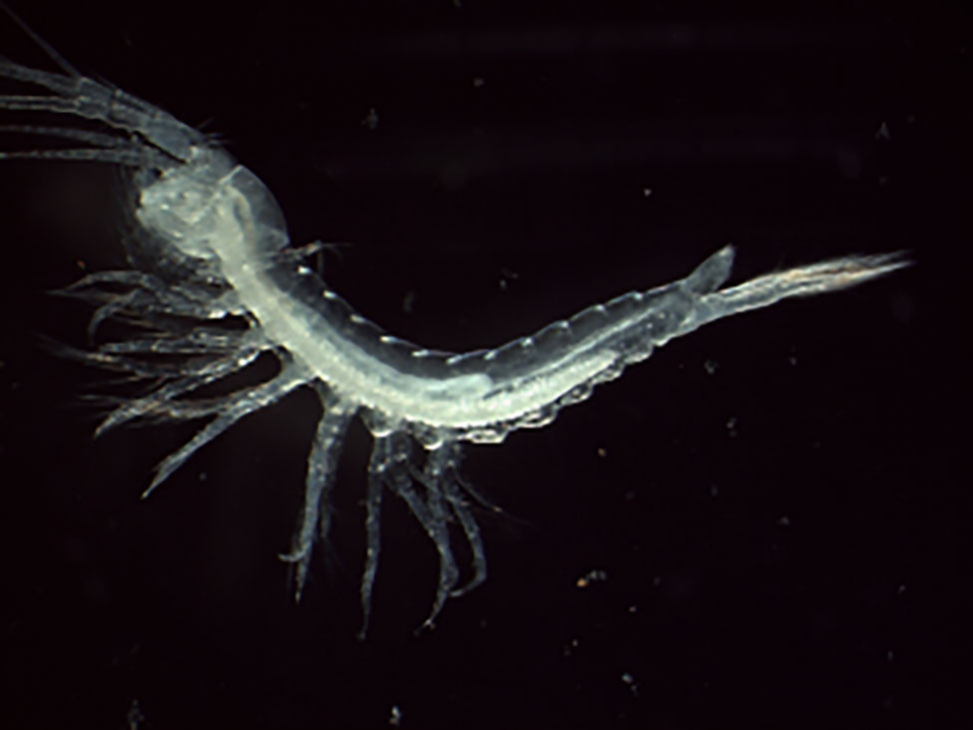
Photograph of a *Tethysbaena scabra* (qmTetScab1) specimen.

*Tethysbaena scabra* (Pretus, 1991) (NCBI:txid203899) is a thermosbaenacean (Crustacea; Multicrustacea; Malacostraca; Eumalacostraca; Peracarida; Thermosbaenacea; Monodellidae), a relict group of peracarid crustaceans characterized by the display in gravid females of a dorsal brood pouch formed by a posterior extension of the carapace ( Figure 1). This species measures 2–3 mm in length and is completely eyeless and depigmented, inhabiting subterranean waters of raised salinity in caves and wells located near the marine coast. It is endemic to the Mediterranean islands of Mallorca and Menorca (Balearic Archipelago). Its feeding habits correspond to those of a particle collector, thriving primarily in the pycnoclines that develop within the water column of anchialine caves, where organic debris, bacteria, and fungi accumulate. There is no available information on genome size and chromosome number in thermosbaenaceans. The closest taxa with known information on genome size ( https://www.genomesize.com, 1C values in pg) are within the peracarid groups Isopoda (1.70-8.60); Amphipoda (0.52-64.62); and Mysida (10.81-12.00).

The genome sequence from *T. scabra* will help to study adaptation to underground environments, particularly anchialine ones, that are characterized by oligotrophy, darkness and salinity. The genome of *T. scabra* was sequenced under the umbrella of the Catalan Initiative for the Earth BioGenome Project (CBP). Here we present a chromosome-level genome assembly for *T. scabra* from Mallorca, Spain, which represents the first reference genome for the order Thermosbaenacea.

## Methods

**Figure 2.**
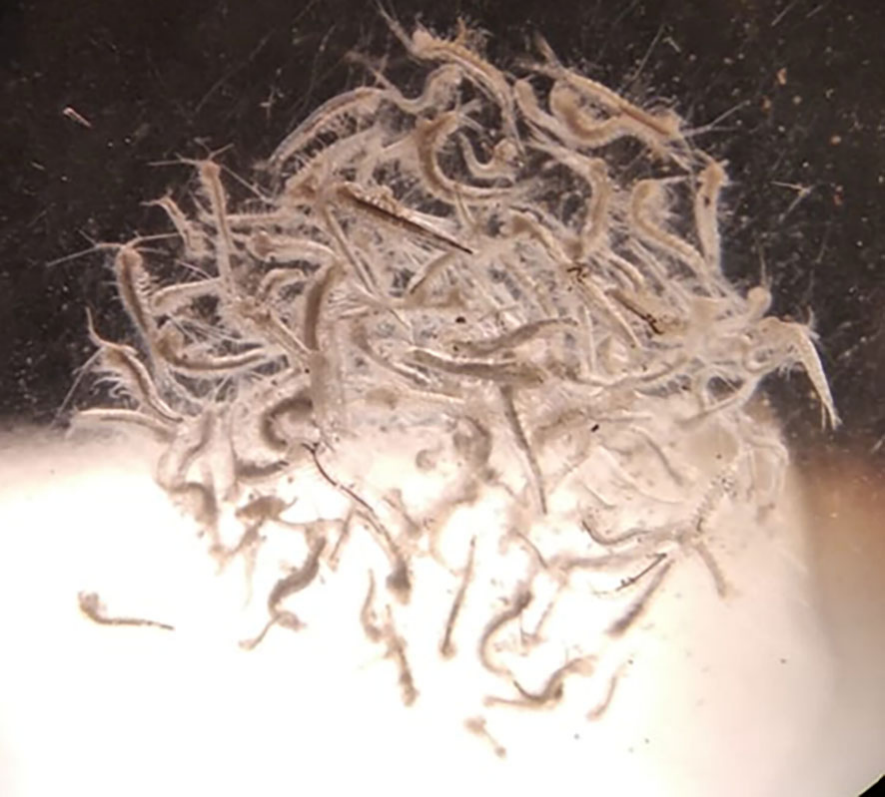
Photograph of *Tethysbaena scabra* specimens under magnification.

Specimens were collected in late Spring 2022 with a modified plankton net from the bottom of a well in an old windmill at Es Pil·larí, Palma, Mallorca, Spain (39.533831, 2.747581). Specimens were sorted out under a stereo-microscope ( Figure 2). Several batches of 20 specimens each were placed in a cryovial for snap-freezing in liquid nitrogen, and ulteriorly sent in dry ice to the sequencing facilities. Specimens were collected and identified by Damià Jaume. Extraction of High Molecular Weight DNA, construction of Pacific Biosciences HiFi circular consensus DNA sequencing libraries, and sequencing on Pacific Biosciences SEQUEL II (HiFi) instrument was performed by Delaware Biotechnology Institute, University of Delaware (DE, USA) using a pool of 20 specimens (Accession number: SAMEA11313135, qmTetScab1). Hi-C data was generated from another pool of 20 individuals from the same collection site (Accession number: SAMEA118091338) using the library preparation Omni-C DNA and sequenced 2 x 150 pb on the Illumina NovaSeq 6000 S4 instrument at the Centre Nacional d’Anàlisi Genòmica (CNAG), Barcelona, Spain.

The genome size was estimated using GenomeScope2 ( Vurture et al., 2017), and diploidy was confirmed with Smudgeplot ( Ranallo-Benavidez et al., 2020). Assembly was conducted using hifiasm ( Cheng et al., 2021) with n_hap=40 (considering diploidy and 20 individuals). Large number of haplotypic duplications presumably caused the high number of specimens used for DNA extraction were withdrawn with purge_dups ( Guan et al., 2020), passing from 2208 to 1272 contigs. Genomic DNA was extracted from individuals whose size is smaller than 5 mm, therefore they were not externally cleaned so it could also contain DNA from microbial and other eukaryote contaminants. Hence, contig sequences from contaminant species were removed from assembly using two bioinformatic tools, Foreign Contamination Screen (FCS, Astashyn et al., 2024), and Whokaryote ( Pronk and Medema, 2022), obtaining 993 contigs. The former achieves this by aligning assemblies, preprocessed to mask repetitive and low-complexity regions, to a curated reference database. The pipeline segments scaffolds into 100-kb subsequences and employs hashed k-mers as alignment seeds. Sequences assigned to taxonomic groups distinct from the query organism (NCBI:txid203899) were then excluded. The latter is a computational tool that differentiates eukaryotic from prokaryotic contig sequences based on fundamental differences in gene structure between the two taxonomic domains. It utilizes a Random Forests approach in combination with Tiara predictions, which incorporate k-mer frequency distributions as classification feature. The assembly was scaffolded with Hi-C data ( Rao et al., 2014) using YaHS ( Zhou et al., 2023), obtaining 821 scaffolds. The assembly was checked for contamination with two rounds of Blobtools, to ensure complete decontamination, obtaining 59 scaffolds. FCS and Whokaryote removed very few sequences compared to BlobToolKit because the first ones only use a close taxon reference, not available in Thermosbaenacea, and gene structure and domains, while the latter is based on several features (GC content, coverage, BUSCO reference, etc.). Curation of contact map was performed using Pretext ( Harry, 2022). A final assembly is obtained with 322 contigs, structured in 23 scaffolds, which present contact patterns in the central regions, suggesting a connection between the scaffolds, which ultimately allows for a total of 17 scaffolds to be obtained. Putative sex chromosomes have not been identified, likely due to the genomic material being sourced from a pool of 20 individuals of unknown sex, and the Hi-C data being derived from a separate pool of specimens. Additionally, the coverage obtained has not been sufficient to deduce sex-linked chromosomes. The genome was analysed within the BlobToolKit environment and BUSCO scores were generated ( Challis et al., 2020). Table 1 list the software tool versions used, where appropriate. To assess the assembly metrics, the k-mer completeness and QV consensus quality values were calculated using Meryl and Merqury ( Rhie et al., 2020).

**Table 1.** Software tools: versions and sources.

Software tool	Version	Source
Blastn	2.12.0+	https://blast.ncbi.nlm.nih.gov/doc/blast-help/downloadblastdata.html
BlobToolKit	4.3.5	https://github.com/blobtoolkit/blobtoolkit
BUSCO	5.5.0	https://gitlab.com/ezlab/busco/-/archive/5.5.0/busco-5.5.0.zip
FCS	0.5.3	https://github.com/ncbi/fcs
GenomeScope2	2.0	https://github.com/tbenavi1/genomescope2.0
Hifiasm	0.20.0-r639	https://github.com/chhylp123/hifiasm
Merqury	1.3	https://github.com/marbl/merqury
Meryl	1.4.1	https://github.com/marbl/meryl
PretextMap	0.1.9	https://github.com/sanger-tol/PretextMap
RepeatMasker	4.1.7	https://github.com/Dfam-consortium/RepeatMasker
RepeatOBServer	1.0	https://github.com/celphin/RepeatOBserverV1
Purge_dups	1.2.5	https://github.com/dfguan/purge_dups
Smudgeplot	0.3.0	https://github.dev/KamilSJaron/smudgeplot
Whokaryote	1.1.2	https://github.com/LottePronk/whokaryote
YaHS	1.2	https://github.com/c-zhou/yahs

The assembly of mitochondrial genome failed using MitoHiFi ( Uliano-Silva et al., 2023), likely due to lack in genome databanks of a mitogenome sequence of sufficiently close taxa. For this reason, sequence contigs were compared with a relaxed BLASTn algorithm against a database built with mitogenome sequences of several peracarid species. The sequence of 30 kb with a positive match was circularized in MitoMaker ( Schomaker-Bastos and Prosdocimi, 2018), and annotated in Mitos2 ( Donath et al., 2019).

Repetitive annotation was performed using RepeatMasker ( Smit et al., 2013–2015) and RepeatOBserver ( Elphinstone et al., 2025). The former tool identifies DNA low complexity regions as well as interspersed repeats. In contrast, RepeatOBserver describes tandem repeats and cluster of transposons found on a chromosome level assembly, based in repeat patterns. In also returns a predicted centromere location for each chromosome.

## Results

**Figure 3.**
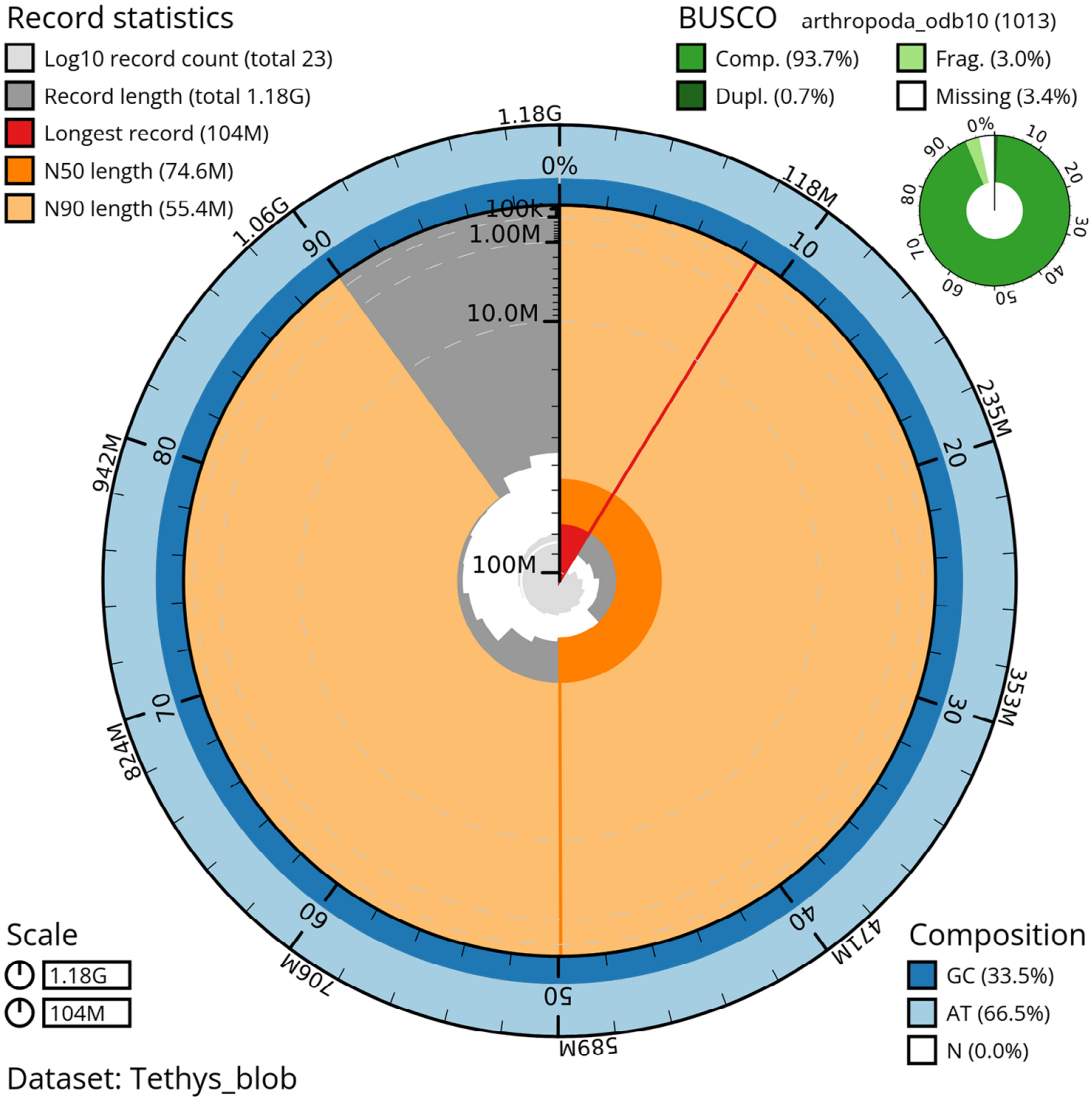
Snailplot of the genome assembly of *Tethysbaena scabra*, qmTetScab1. This snailplot generated by BlobToolKit displays several metrics, including the longest scaffold, N50, and BUSCO gene completeness, among others. The main plot is segmented into 50 bins, ordered by size around the circumference, with each bin representing 2% of the 1.18 Gbp assembly. Scaffold length distribution is shown in dark grey, with the plot radius scaled to the length of the longest scaffold in the assembly (104 Mbp). Orange and light-orange arcs indicate the N50 and N90 scaffold lengths (74.6 Mbp and 55.4 Mbp, respectively). A pale grey spiral illustrates the cumulative scaffold count on a log scale, with white scale lines marking successive orders of magnitude. The blue and pale-blue areas along the plot's outer edge depict the GC, AT, and N content distribution across these bins. A summary of the BUSCO results appears in the figure’s top right corner.

**Figure 4.**
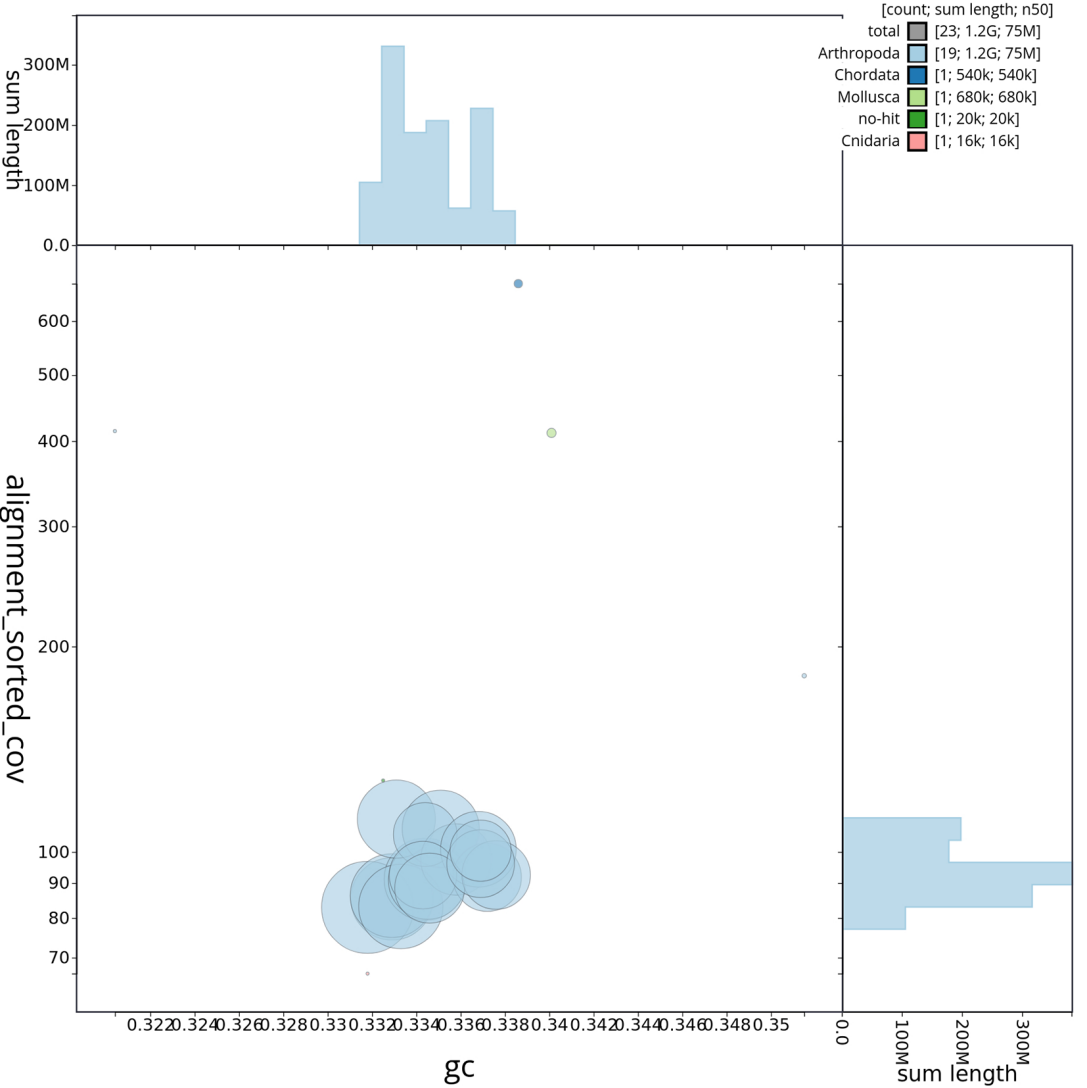
Genome assembly of *Tethysbaena scabra,* qmTetScab1.1: BlobToolKit GC-coverage plot. Scaffolds are shown by phylum. Circles are sized in proportion to scaffold length. Histograms show the distribution of scaffold length sum along.

**Figure 5.**
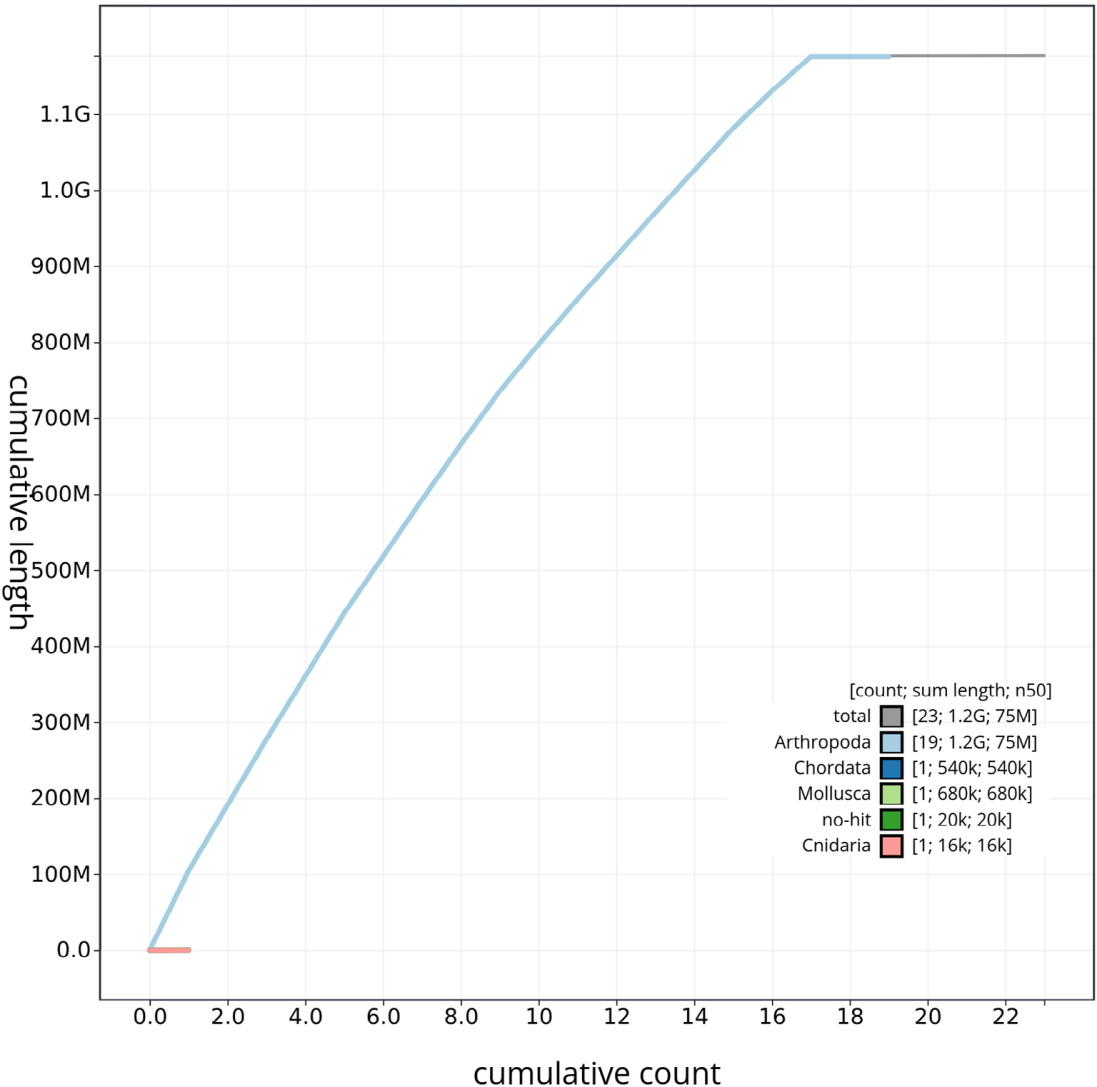
Genome assembly of *Tethysbaena scabra*: BlobToolKit cumulative sequence plot, qmTetScab1.1. The gray line represents the cumulative length of all scaffolds, while the colored lines indicate the cumulative lengths of scaffolds assigned to each individual phylum.

**Figure 6.**
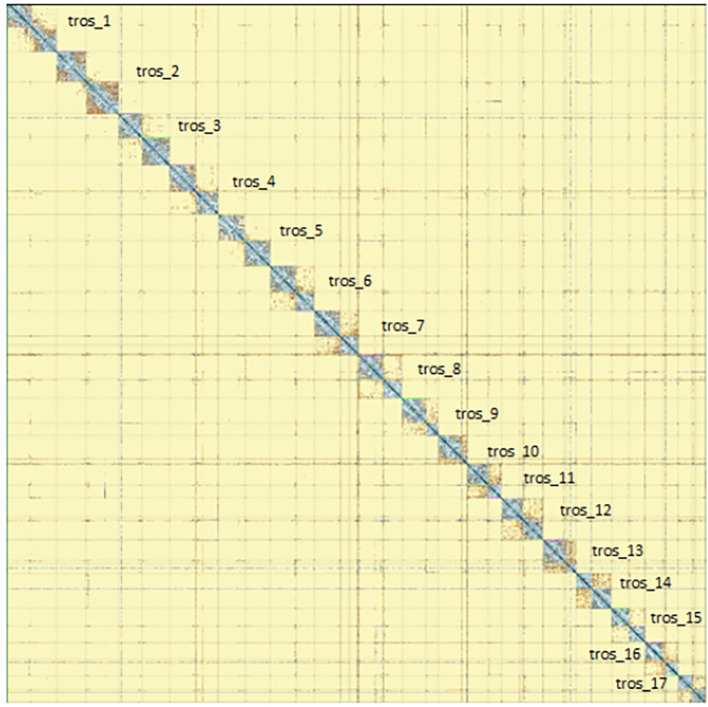
Genome assembly of *Tethysbaena scabra,* qmTetScab1: Hi-C contact map of assembly, visualised using PretextMap. Chromosomes are shown as they appear in PretextMap, not by size order.

The genome sequence was obtained from a DNA pool of 20 specimens of *T. scabra* for HiFi data, plus another identical pool for Hi-C data, from individuals collected in a well in Es Pil·larí, Palma, Mallorca, Spain. Two Pacific Biosciences sequencing cells yielded a total of 63.5 giga bases of high-fidelity (HiFi) long reads with a N50 of 13,270 bp, achieving a coverage of 53.8X. Afterward, primary contig assemblies were scaffolded using 73.9 Gb of paired-end Illumina reads derived from chromosome conformation Hi-C data. Manual curation corrected 39 misassemblies, including missing joins and missjoins, resulting in a 0.28% reduction in the total assembly length, a 61.02% decrease in scaffold count, and an 89.99% increase in scaffold N50. The final genome assembly spans 1.18 Gb across 23 scaffolds, with a scaffold N50 of 74.6 Mb ( Figure 3, Table 2). GC-coverage ( Figure 4) and cumulative sequence plots ( Figure 5) from BlobToolKit showed minimal parameter variation with few outliers, and only a very low fraction of sequences failed to match Arthropoda ones deposited in databases. Most of the assembly sequence (99.2%) has been mapped to the final chromosomes. The final assembly sequence confirmed by Hi-C data was assigned to 17 chromosomal-level scaffolds that are designated as they appear in the PretextMap ( Figure 6; Table 3). The assembly has a BUSCO v5.5.0 ( Manni et al., 2021; Simão FA et al., 2015) completeness of 94.7% (single 93.7%, duplicated 0.7%) using the arthropoda_odb10 reference set. The mitochondrial genome contig can be found within the multifasta file of the genome submission.

**Table 2.** Genome data for *Tethysbaena scabra*, qmTetScab1.1. Assembly metrics benchmarks are adapted from the 6.C.Q40 of Earth Biogenome Project from ( Lawniczak et al., 2022). BUSCO scores based on the arthropoda_odb10 BUSCO set using v5.5.0. C = complete, [S = single copy, D = duplicated], F = fragmented, M = missing, n = number of orthologues in comparison.

Project accession data		
Assembly name	*Tethysbaena scabra*	
Assembly accession	GCA_964277195	
Accession of alternate haplotype	-	
Span (Mb)	1200	
Number of contigs	322	
Contig N50 length (Mb)	6.1Mb	
Number of scaffolds	23	
Scaffold N50 length (Mb)	74.5Mb	
Longest scaffold (Mb)	104.45Mb	
‍Gaps (bp)	299 standardized 100 bp gaps	
**Assembly metrics**		** Benchmark**
Consensus quality (QV)	50.41	≥40
K-mer completeness	92.5	≥90
Busco	C:93.7%[S:93,D:0.7%], F:3%,M:3.4%,n:1,013	C ≥90%, D <5%
Percentage of assembly mapped to chromosomes	99.2%	≥90%
Organelles	MT	Complete single alleles

**Table 3.** Chromosomal pseudomolecules in the genome assembly of *Tethysbaena scabra.* https://www.ebi.ac.uk/ena/browser/view/GCA_964277195.1?show=chromosomes.

Accession	Name	Length (Mb)	GC%
OZ195310	tros_1	83.11	33.29
OZ195311	tros_2	104.46	33.18
OZ195312	tros_3	85.72	33.29
OZ195313	tros_4	82.79	33.44
OZ195314	tros_5	87.20	33.33
OZ195315	tros_6	74.56	33.45
OZ195316	tros_7	74.67	33.31
OZ195317	tros_8	72.98	33.51
OZ195318	tros_9	61.70	33.58
OZ195319	tros_10	49.14	33.44
OZ195320	tros_11	56.67	33.72
OZ195321	tros_12	70.05	33.68
OZ195322	tros_13	55.35	33.43
OZ195323	tros_14	59.37	33.46
OZ195324	tros_15	57.12	33.76
OZ195325	tros_16	55.68	33.69
OZ195326	tros_17	45.10	33.69
OZ195327	MT	0.016	32.04

The genome annotation was assessed using BUSCO obtaining: C:93.1% [S:73.2%, D:19.9%], F:2.2%, M:4.7%, also 27,004 transcripts and 22,834 genes. RNAQuast has been performed to check the average alignment length, being 1248.6 bp. Repetitive regions are summarized in Table 4.

**Table 4.** Summary of the repetitive elements found by RepeatMasker in the genome of *Tethysbaena scabra*, qmTetScab1.1.

		Number of elements	Length occupied	%
SINEs:		3,285	217,586 bp	0.02%
	ALUs	7	499 bp	0.00%
	MIRs	381	30,645 bp	0.00%
LINEs:		100,876	97,666,309 bp	8.30%
	LINE1	3,138	378,718 bp	0.03%
	LINE2	47,591	44,895,798 bp	3.81%
	L3/CR1	49,210	52,023,230 bp	4.42%
LTR elements:		1,726	541,618 bp	0.05%
	ERVL	80	10,534 bp	0.00%
	ERVL-MaLRs	118	12,225 bp	0.00%
	ERV_classI	1,224	337,524 bp	0.03%
	ERV_classII	46	4,692 bp	0.00%
DNA elements:		39,909	19,071,121 bp	1.62%
	hAT-Charlie	20,903	9,453,122 bp	0.80%
	TcMar-Tigger	3,285	1,466,820 bp	0.12%
Unclassified		20	3,649 bp	0.00%
Total	Interspersed		117,500,283 bp	9.98%
	Small RNA	1,757	176,391 bp	0.01%
Satellites:		94	13,096 bp	0.00%
	Simple repeats	552,457	26,333,387 bp	2.24%
	Low complexity	71,177	3,444,953 bp	0.29%

### Ethics and consent

Ethical approval and consent were not required.

## Author contributions

Conceptualization (JP, CJ, DJ, JAJR), Data Curation (KDSA, LTL, JP), Formal Analysis (LTL, KDSA, JP), Funding Acquisition (JAJR, JP), Resources (DJ), Writing – Original Draft Preparation (LTL, KDSA, JP), and Writing – Review & Editing (all).

## Data Availability

The *Tethysbaena scabra* genome project is integrated into the Catalan Initiative for the Earth BioGenome Project (CBP), and all raw data and assembly were deposited in European Nucleotide Archive: *Tethysbaena scabra.* Accession number PRJEB61927; https://identifiers.org/ena.embl/PRJEB61927. Raw data and assembly accession identifiers are reported in Table 3.
