# Supplementary material for: The genome sequence of Tethysbaena scabra (Pretus, 1991), the first known in the peracarid crustacean order Thermosbaenacea
Source: F1000Res. 2025 Sep 19;14:293. Originally published 2025 Mar 14. [Version 3] doi: 10.12688/f1000research.161461.3 (PMC12481149; doi:10.12688/f1000research.161461.3)
Supplement: Peer review history

# Reviewer report: Reviewer response for version 3

**DOI:** 10.5256/f1000research.188221.r415498

**Published:** 2025-09-29

**Authors:** Martin Schwentner

**Affiliations:** 1 Naturhistorisches Museum Vienna (Austria), Vienna, Austria

I think the manuscript is ready for acceptance, the authors have responded to all raised issues and altered all relevant sections

Are the datasets clearly presented in a usable and accessible format, and the assembly and annotation available in an appropriate subject-specific repository?

Partly

Are sufficient details of the sequencing and extraction, software used, and materials provided to allow replication by others?

Yes

Are the rationale for sequencing the genome and the species significance clearly described?

Yes

Are the protocols appropriate and is the work technically sound?

Yes

Reviewer Expertise:

genomics, systematics, evolutionary research, crustacea

I confirm that I have read this submission and believe that I have an appropriate level of expertise to confirm that it is of an acceptable scientific standard.

# Reviewer report: Reviewer response for version 2

**DOI:** 10.5256/f1000research.183424.r406386

**Published:** 2025-09-04

**Authors:** Martin Schwentner

**Affiliations:** 1 Naturhistorisches Museum Vienna (Austria), Vienna, Austria

The authors present the first genome of the thermosbaenacean, which will be an important resource for future research. The overall manuscript is well written and structured and the methods and results are appropriate and well presented. 

 I have a few comments that will help to clarify some issues.

 1. The authors described in the Amendments that they now report 299 standardized 100 bp gaps, but I could not find this information in the actual manuscript.

 2. I tried to download the genome. Maybe I have missed it, but I could not find the whole genome (I was only able to download the first scaffold) and could not find a gene file or similar. Please make sure that all data is available

 3. The contamination check with Blobtools had a strong impact as it removed ~770 of the 820 scaffolds. Due to its impact, it would be very important and helpful if the actual value used were described. Currently only the metrices are named (GC, coverage, BUSCO), but not the specific values or ranges employed.

 4. I find a bit difficult to follow the reported numbers of scaffolds and contigs, and if I understand the text correctly, the final number of contigs and scaffold does not quite match those from the beginning. 993 contigs were assembled into 821 scaffold (thus most scaffolds include only one contig). Only 59 scaffolds were retained after Blobtools filtering and the final set is 322 contigs in 23 scaffolds. That should not be possible, even the 59 scaffolds after Blobtools should not hold more then ~200 contigs. Maybe I did not fully understand the numbers, the authors should make this procedure clearer

Are the datasets clearly presented in a usable and accessible format, and the assembly and annotation available in an appropriate subject-specific repository?

Partly

Are sufficient details of the sequencing and extraction, software used, and materials provided to allow replication by others?

Yes

Are the rationale for sequencing the genome and the species significance clearly described?

Yes

Are the protocols appropriate and is the work technically sound?

Yes

Reviewer Expertise:

genomics, systematics, evolutionary research, crustacea

I confirm that I have read this submission and believe that I have an appropriate level of expertise to confirm that it is of an acceptable scientific standard, however I have significant reservations, as outlined above.

# Response

**Published:** 2025-09-09

**Authors:** Joan Pons

**Affiliations:** Animal&Microbial Biodiversity, Institut Mediterrani d'Estudis Avancats, Esporles, Illes Balears, Spain

Here are the response of each question:

*1. The authors described in the Amendments that they now report 299 standardized 100 bp gaps, but I could not find this information in the actual manuscript.*

 The information about the 299 standardized 100 pb gaps was included in Table 2. The new version also includes that information in the main text: “The contact map was curated using Pretext (Harry, 2022), which suggested connections between scaffolds and reduced the final assembly from 59 to 17 scaffolds, while retaining 229 scaffolds of unknown size (represented as 100 consecutive Ns in the FASTA file).”.

*2. I tried to download the genome. Maybe I have missed it, but I could not find the whole genome (I was only able to download the first scaffold) and could not find a gene file or similar. Please make sure that all data is available*

 The link in the manuscript https://identifiers.org/ena.embl/PRJEB61927 points to https://www.ebi.ac.uk/ena/browser/view/PRJEB61927 in ENA from where user can go to right panel and click on Related ENA Records and find links to the results of the project:

 Result Count

 Experiment (PACBIO, HiC, and RNAseq reads) 3

 Assembly 1

 Genome assembly contig set 1

 Sequence (By Chromosome) 18

 Run ( PACBIO, HiC, and RNAseq reads) 3

 Raw data, PACBIO, HiC and RNAseq reads, are also available downloaded from NCBI under the Bioprojet PRJEB61927

*3. The contamination check with Blobtools had a strong impact as it removed ~770 of the 820 scaffolds. Due to its impact, it would be very important and helpful if the actual value used were described. Currently only the metrices are named (GC, coverage, BUSCO), but not the specific values or ranges employed.*

 GC, length, and coverage after cut-off in Blobtools are indicated in Figure 4. Here we summarize the cut-off filter values implemented in the two rounds of Blobtools:

 Fisrt round in Blobtoolkit:

 GC_MIN = 0.320

 GC_MAX = 0.351

 SORTED_ALIGNMENT_COVERAGE_MIN = 36.320

 SORTED_ALIGNMENT_COVERAGE_MAX = 680.661

 LENGTH_MIN = 10000

 Second round in Blobtoolkit:

 GC_MIN = 0.330

 GC_MAX = 0.340

 SORTED_ALIGNMENT_COVERAGE_MIN = 50.000

 SORTED_ALIGNMENT_COVERAGE_MAX = 170.000

 LENGTH_MIN = 10000

 We replaced the results shown in Figure 4, which correspond to the first round of filtering in BlobTools, with those obtained after the second round.

*4. I find a bit difficult to follow the reported numbers of scaffolds and contigs, and if I understand the text correctly, the final number of contigs and scaffold does not quite match those from the beginning. 993 contigs were assembled into 821 scaffold (thus most scaffolds include only one contig). Only 59 scaffolds were retained after Blobtools filtering and the final set is 322 contigs in 23 scaffolds. That should not be possible, even the 59 scaffolds after Blobtools should not hold more then ~200 contigs. Maybe I did not fully understand the numbers, the authors should make this procedure clearer*

 Our previous explanation was confusing, so we provide a new wording in the main text: “The contact map was curated using Pretext (Harry, 2022), which suggested connections between scaffolds and reduced the final assembly from 59 to 17 scaffolds, while retaining 229 scaffolds of unknown size (represented as 100 consecutive Ns in the FASTA file).

# Reviewer report: Reviewer response for version 2

**DOI:** 10.5256/f1000research.183424.r396911

**Published:** 2025-08-22

**Authors:** Pascal Angst

**Affiliations:** 1 University of Basel, Basel, Switzerland

The revised manuscript effectively addresses the comments I made as a reviewer. I appreciate the improvements made and the attention given to the issues I raised. Most of the revisions are clear and well implemented. However, I would be interested in receiving some brief clarification on a few of the changes. Could the authors please elaborate on the following points:

  

- The accession added, “SAMEA11313135”, is titled “COVID-HUB-PL deep NGS sequencing of SARS-CoV-2 genomes” in the ENA. Should this be “SAMEA113414145”? – For “SAMEA118091338”, the associated Hi-C data seems to be unavailable on the ENA website.
- “In also returns a predicted centromere location for each chromosome.” “It also […]”? Further, did you find any centromeres or centromere-characteristic repeats?
- “The genome annotation was assessed using BUSCO obtaining: C:93,1% [S:73,2%, D:19,9%], F:2,2%, M:4,7%, also 27004 transcripts and 22834 genes. RNAQuast has been performed to check the average alignment length, being 1248,6bp.” This is the first time that transcripts, genes, and RNA are mentioned. Is this related to the RNA-seq in PRJEB61927? Was gene annotation performed? If so, how was it done, and where can the annotations be found?

Are the datasets clearly presented in a usable and accessible format, and the assembly and annotation available in an appropriate subject-specific repository?

Partly

Are sufficient details of the sequencing and extraction, software used, and materials provided to allow replication by others?

Partly

Are the rationale for sequencing the genome and the species significance clearly described?

Partly

Are the protocols appropriate and is the work technically sound?

Partly

Reviewer Expertise:

Genome assembly and annotation | Wet Lab (DNA/RNA extraction and library preparation) | Short- and Long-read sequencing technologies | Evolutionary Biology | Population Genetics | Metapopulation Ecology | Host-Parasite Interactions | Genome Evolution | Environmental Science

I confirm that I have read this submission and believe that I have an appropriate level of expertise to confirm that it is of an acceptable scientific standard.

# Response

**Published:** 2025-09-09

**Authors:** Joan Pons

**Affiliations:** Animal&Microbial Biodiversity, Institut Mediterrani d'Estudis Avancats, Esporles, Illes Balears, Spain

We reply to each question:

*1) The accession added, “SAMEA11313135”, is titled “COVID-HUB-PL deep NGS sequencing of SARS-CoV-2 genomes” in the ENA. Should this be “SAMEA113414145”? – For “SAMEA118091338”, the associated Hi-C data seems to be unavailable on the ENA website.*

 The biosample to obtain PACBIO data is SAMEA113414145. Sorry for the mistake. We fix the error on the new version. The biosample for HiC data is SAMEA118091338.

 The link in the manuscript https://identifiers.org/ena.embl/PRJEB61927 points to https://www.ebi.ac.uk/ena/browser/view/PRJEB61927 in ENA. Then, in right panel the user can click on Related ENA Records and find links to all results of the project:

 Result Count

 Experiment (PACBIO, HiC, and RNAseq reads) 3

 Assembly 1

 Genome assembly contig set 1

 Sequence (By Chromosome) 18

 Run ( PACBIO, HiC, and RNAseq reads) 3

 Raw data, PACBIO, HiC and RNAseq reads, are also available downloaded from NCBI under the Bioprojet PRJEB61927

*2) “In also returns a predicted centromere location for each chromosome.” “It also […]”? Further, did you find any centromeres or centromere-characteristic repeats?*

 We did not analyze the sequences of centromere repeats. We just annotated them.

*3) “The genome annotation was assessed using BUSCO obtaining: C:93,1% [S:73,2%, D:19,9%], F:2,2%, M:4,7%, also 27004 transcripts and 22834 genes. RNAQuast has been performed to check the average alignment length, being 1248,6bp.” This is the first time that transcripts, genes, and RNA are mentioned. Is this related to the RNA-seq in PRJEB61927? Was gene annotation performed? If so, how was it done, and where can the annotations be found?*

 We performed a preliminary of the RNA-seq data from another sample (biosample SAMEA117791495) to obtain a preliminary number of coding genes. The workflow consists of several steps. In brief, Illumina paired-end adapters (Nextera PE) were removed from the raw reads using Trimmomatic v0.39. Next, the reference genome assembly was soft-masked with dustmasker, and an index was generated with hisat2-build. The trimmed reads were then aligned to the indexed genome using hisat2, and the resulting alignments were processed with samtools. Finally, gene annotation was performed with BRAKER v3, using a protein database retrieved from OrthoDB as external evidence. RNA-seq data is available in the project ( https://identifiers.org/ena.embl/PRJEB61927 ). Here is a direct link

https://www.ebi.ac.uk/ena/browser/view/ERX14107725 . We are still working to make a full annotation in the near future once new funding is obtained.

# Reviewer report: Reviewer response for version 1

**DOI:** 10.5256/f1000research.177497.r376178

**Published:** 2025-04-16

**Authors:** Pascal Angst

**Affiliations:** 1 University of Basel, Basel, Switzerland

This genome note presents the genome of *Tethysbaena scabra*. The authors sampled two pools of specimens for sequencing using PacBio HiFi and Hi-C technologies. They used latest software for assembly of sequencing reads and for assessing the assembly’s quality, completeness, and contamination level. They discussed potential sources of contamination and they separated target versus non-target, contaminant contigs based on specialized software. Generally, this article is sound, but I identified a few inconsistencies and lack of detail which I would like the authors to address. I also expected more details on the sequence (variation) of the genome from a genome note, e.g., a summary of the repeat content and other annotations.

 Details on the mandatory reviewer questions:

- The main rationale presented for sequencing the genome was the Catalan Initiative for the Earth BioGenome Project (CBP). It would thus be nice to have a short explanation of what that is and what its significance is.
- The protocols and the work seem technically sound but would profit from more details. For example, it is mentioned that pools of specimens were used for sequencing but not why this was done. Clearly, sequencing a single individual would be preferable. Related to this, the methods state that two pools of 20 specimens were sampled. However, the results state that “identical” pools were used. The word identical is confusing in that context, because in the methods the pools are described as separate pools. Also, on NCBI, there is only one BioSample (a batch of 20 individuals) registered and is linked to the Illumina and the PacBio sequencing, which is not what is described in the article. The BioSample should either be a batch of 40 individuals or there should be two BioSamples of 20 individuals each.
  Details on DNA extraction and library preparations are missing. To reproduce the work or to apply it to other systems, it is necessary to know what kits, reagents, and protocols were used.
- To replicate the assembly process and subsequent assembly modification the parameters used for the software are missing. Especially, what parameters were used for hifiasm? Given it was not designed for assembly of multiple specimens’ genomes, were the parameters adjusted accordingly? It seems hifiasm had issues in the collapsing step since the authors applied purge_dups, which lead to a great reduction in the number of contigs. It is normally not desired to purge haplotigs from hifiasm assemblies and if applied does not result in such a drastic reduction in the number of contigs. I missed discussion of all of this.
  “individuals that were not externally cleaned so it could also contain DNA from microbial and other eukaryote contaminants”. Why were the individuals not cleaned before assembly despite that this is known to cause assembly issues?
  It is not clear how many contigs remained after each step in the methods. What was the number of contigs after Hi-C scaffolding? In the method is says 821. It also says 59 contigs were obtained after applying BlobToolKit. Are the other 762 (821 - 59) contigs from contaminants? How does that align with the 322 contigs mentioned in Table 2 and the 17 scaffolds mentioned in the Results?
  "The coverage obtained has not been sufficient to deduce sex-linked chromosomes". Would this be a sensible analysis given the pools of specimens? Why is 53.8x coverage not enough? Is this the haploid sequence coverage? From Figure 4, the coverage seems twice as high.
- The genome is available from NCBI. However, there are no annotations provided. At least a description of the repetitive content would be valuable. Repetitive content seems to have been assessed since assemblies were "preprocessed to mask repetitive and low-complexity regions".

Additional aspects:

- The completeness of the assembly was assessed using BUSCOs and k-mers. Given the chromosome-level assembly, it would be valuable to describe the sequence content and arrangement, and the structural variation. For example, what is the telomeric end repeat motif; what characterizes the centromeres (GC content, sequence content, repeat content); what is the distribution of repeat versus genic content?
- It would be important to know the average read lengths or read length N50s.
- The keywords should include the full species name.
- Are there assembly gaps? What is their size?
- There is no phylogenetic analysis. I suggest including one or refer to a previous solid (whole genome) phylogeny.

Are the datasets clearly presented in a usable and accessible format, and the assembly and annotation available in an appropriate subject-specific repository?

Partly

Are sufficient details of the sequencing and extraction, software used, and materials provided to allow replication by others?

Partly

Are the rationale for sequencing the genome and the species significance clearly described?

Partly

Are the protocols appropriate and is the work technically sound?

Partly

Reviewer Expertise:

Genome assembly and annotation | Wet Lab (DNA/RNA extraction and library preparation) | Short- and Long-read sequencing technologies | Evolutionary Biology | Population Genetics | Metapopulation Ecology | Host-Parasite Interactions | Genome Evolution | Environmental Science

I confirm that I have read this submission and believe that I have an appropriate level of expertise to confirm that it is of an acceptable scientific standard, however I have significant reservations, as outlined above.

# Response

**Published:** 2025-06-06

**Authors:** Joan Pons

**Affiliations:** Animal&Microbial Biodiversity, Institut Mediterrani d'Estudis Avancats, Esporles, Illes Balears, Spain

1.  The main rationale presented for sequencing the genome was the Catalan Initiative for the Earth BioGenome Project (CBP). It would thus be nice to have a short explanation of what that is and what its significance is. ** *We added additional information as suggested. “The Catalan Biogenome is EBP-affiliated project network with the objective of sequencing the genome of more than 40000 eukaryotic species living in the Catalan Linguistic Area (such as Balearic Islands)”.*
2.  The protocols and the work seem technically sound but would profit from more details. For example, it is mentioned that pools of specimens were used for sequencing but not why this was done. ** *We agree that our wording was confusing so we rewrote the text to clarify the issue “Extraction of high molecular weight DNA, construction of Pacific Biosciences HiFi circular consensus DNA sequencing libraries, and sequencing on Pacific Biosciences SEQUEL II (HiFi) instrument was performed by Delaware Biotechnology Institute, University of Delaware (DE, USA) using a pool of 20 specimens (Accession number: SAMEA113414145 qmTetScab1). Hi-C data was generated from another pool of 20 individuals from the same collection site (Accession number: SAMEA118091338) using the library preparation Omni-C DNA and sequenced 2 x 150 pb on the Illumina NovaSeq 6000 S4 instrument at the Centre Nacional de Seqüenciació Genòmica (CNAG), Barcelona, Spain." *
3. Details on DNA extraction and library preparations are missing. To reproduce the work or to apply it to other systems, it is necessary to know what kits, reagents, and protocols were used. ** *For the HIFI sequencing, a DNA library was prepared using the PacBio SMRTbell Express Template Prep Kit 2.0* *(Pacific Biosciences of California, CA, USA)* *following the official protocol. Approximately 300 ng of high-quality genomic DNA was sheared to ~15–20 kb, repaired, and ligated with SMRTbell adapters to create circular molecules. The library was size-selected to remove fragments smaller than 1,000 bp, purified with AMPure PB beads, and quality-checked using Qubit and TapeStation. Finally, it was sequenced on the PacBio Sequel II platform in CCS mode to generate highly accurate HiFi reads suitable for genome assembly and analysis. These previous steps were performed in the University of Delaware (USA).* ** *Hi-C libraries were prepared using the Omni-C Dovetail protocol (Cantara Bio, CA, USA). Briefly, chromatin was extracted from frozen tissue and crosslinked with formaldehyde to preserve the native three-dimensional genome architecture by stabilizing DNA-protein and DNA-DNA interactions within the nucleus. The crosslinked chromatin was then fragmented using DNase I, and spatially proximal DNA ends were ligated to capture physical interactions between genomic regions, providing long-range linkage information useful for validating and scaffolding genome assemblies. Hi-C library preparation and sequencing were performed at the Centre Nacional d’Anàlisi Genòmica (CNAG), Barcelona, Spain.*
4. To replicate the assembly process and subsequent assembly modification the parameters used for the software are missing. Especially, what parameters were used for hifiasm? Given it was not designed for assembly of multiple specimens’ genomes, were the parameters adjusted accordingly? It seems hifiasm had issues in the collapsing step since the authors applied purge_dups, which lead to a great reduction in the number of contigs. It is normally not desired to purge haplotigs from hifiasm assemblies and if applied does not result in such a drastic reduction in the number of contigs. I missed discussion of all of this. ** *The number of haplotypes was considered using the --nhap parameter in hifiasm.* *Given that* *our species is presumably diploid, as estimated by Smudgeplot, and* *the sequencing* *pool i* *ncluded* *20 individuals,* *the* *parameter was* *set to* *nhap=40.* *However, d* *ue to the* *presence of multiple* *individuals, hifiasm* *may struggle* *to accurately resolve haplotypes, which may necessitate the use of* *purge_dups* *to remove redundant contigs.* ** *We suspect that most of the duplications are due to the DNA being sourced from a pool of 20 individuals, as a single individual did not provide enough material to construct a HiFi library. We clarify this question in the main text “The genome size was estimated using GenomeScope2 (Vurture et al., 2017), and diploidy was confirmed with Smudgeplot (Ranallo-Benavidez et al., 2020). Assembly was conducted using hifiasm (Cheng et al., 2021) with n_hap=40 (considering diploidy and 20 individuals). Larger number of Haplotypic duplications presumably caused by the high number of specimens used for DNA extraction were withdrawn with purge_dups (Guan et al., 2020), passing from 2208 to 1272 contigs". We would like to point out that final genome size after purging duplicates and removing contamination matched the size initially predicted by Genomescope.*
5.  “individuals that were not externally cleaned so it could also contain DNA from microbial and other eukaryote contaminants”. Why were the individuals not cleaned before assembly despite that this is known to cause assembly issues? ** Specimens were isolated from well water and individually collected to minimize contamination from other macroscopic species. However, this approach did not prevent contamination by microscopic organisms.
6.  It is not clear how many contigs remained after each step in the methods. What was the number of contigs after Hi-C scaffolding? In the method is says 821. It also says 59 contigs were obtained after applying BlobToolKit. Are the other 762 (821 - 59) contigs from contaminants? How does that align with the 322 contigs mentioned in Table 2 and the 17 scaffolds mentioned in the Results? ** *We rewrote the text to be more sound and included a new sentence in the Methods section.*
7.  "The coverage obtained has not been sufficient to deduce sex-linked chromosomes". Would this be a sensible analysis given the pools of specimens? Why is 53.8x coverage not enough? Is this the haploid sequence coverage? From Figure 4, the coverage seems twice as high. ** *Several factors hindered the identification of sex chromosomes in our diploid species. Most prominently, the characteristic haploid coverage pattern typically associated with sex chromosomes was absent. Furthermore, the genome assembly and scaffolding were performed using two separate DNA pools without prior knowledge of the individuals’ sex, complicating the detection of sex-specific sequences. In addition, the lack of biological information on the species and genus—particularly whether sex determination is chromosomal or genetic—further limits the applicability of standard methods for identifying sex chromosomes.*
8. The genome is available from NCBI. However, there are no annotations provided. At least a description of the repetitive content would be valuable. Repetitive content seems to have been assessed since assemblies were "preprocessed to mask repetitive and low-complexity regions". ** *We added the annotation for the repetitive sequences as requested.*
9. The completeness of the assembly was assessed using BUSCOs and k-mers. Given the chromosome-level assembly, it would be valuable to describe the sequence content and arrangement, and the structural variation. For example, what is the telomeric end repeat motif; what characterizes the centromeres (GC content, sequence content, repeat content); what is the distribution of repeat versus genic content? ** We present a new table summarizing the chromosomal location, composition, and sequences of the repetitive DNA elements.
10.  It would be important to know the average read lengths or read length N50s. ** *The read length N50 of PacBio raw reads has been added to the results section. *
11. The keywords should include the full species name. ** *The species name has been added to the keywords section. *
12. Are there assembly gaps? What is their size? ** The final assembly contains 299 gaps, each 100 bp in length. This is due to the default behavior of tools such as Hifiasm, YAHS, and PretextMap, which insert standardized 100 bp gaps when the true gap size cannot be determined.
13. There is no phylogenetic analysis. I suggest including one or refer to a previous solid (whole genome) phylogeny. ** *We appreciate the suggestion to include a phylogenetic analysis. However, given the current lack of available genomic data from other species in the Pericarid crustacean order, we believe that conducting a robust phylogenetic analysis at this stage would not be sufficiently reliable. We agree that such an analysis would be highly valuable, particularly once more genomic data from related taxa becomes available, and it is a future goal of our research group.*
